# Cognitive Performance During Night Work in the Cold

**DOI:** 10.3389/fphys.2021.768517

**Published:** 2021-12-03

**Authors:** Hilde Færevik, Jakob Hønborg Hansen, Øystein Wiggen, Mariann Sandsund

**Affiliations:** ^1^SINTEF Digital, Department of Health Research, Trondheim, Norway; ^2^Wood Group, Sandefjord, Norway

**Keywords:** day/night work, cold environment, cognitive performance, temperature regulation, sleepiness, cortisol, protective clothing

## Abstract

**Objective:** The objective of this study was to investigate how night work at low ambient temperatures affects cognitive performance (short-term memory and reaction time), skin- and core temperature, thermal comfort, sleepiness, and cortisol. We hypothesized that cognitive performance is reduced at night compared with daytime and worsened when exposed to low ambient temperatures.

**Method:** Eleven male subjects were recruited to perform three tests in a climatic chamber at night and daytime: Night –2°C, Night 23°C and Day 23°C. Each test lasted 6 h. Cognitive performance (short-term memory and reaction time), skin- and core temperature, thermal sensation and comfort, cortisol levels and sleepiness were measured during the tests.

**Results:** A lower mean skin temperature and corresponding lower thermal sensation were observed at Night –2°C compared to Day 23°C and Night 23°C. Night work caused increased sleepiness and lower cortisol levels, but was not affected by changes in ambient temperatures, thermal comfort, or skin temperatures. There was no effect of either day/night work nor ambient temperature on the short-term memory or reaction time test.

**Conclusion:** Lower skin- and core temperature were observed at night when exposed to low ambient temperature (–2°C), but there was no effect on short-term memory or reaction time. Increased sleepiness and lower cortisol levels were observed at night compared to daytime and was not influenced by low ambient temperature at night. The result from this study suggests that cognitive performance (short-term memory and reaction time) is not adversely affected by night work when exposed to low ambient temperatures if adequate protective clothing is worn.

## Introduction

Cold exposure is experienced in many occupational and leisure settings and humans exhibit a range of physiological responses when exposed to cold environments. This includes changes in skin- and core- body temperature inducing e.g., cutaneous vasoconstriction to decrease heat loss and shivering thermogenesis which increase metabolic heat production to maintain thermal balance (Castellani and Young, 2016). Exposure to low ambient temperatures may also be associated with declines in cognitive functioning. Both increase and decrease in accuracy and efficiency on cognitive tests assessing vigilance, reasoning and memory have been found in laboratory settings (Palinkas, 2001; Pilcher et al., 2002; Hancock et al., 2007). The few observational studies that have examined the association between and cognition report a “U” shaped relationship of temperature and performance (Ramsey, 1995; Dai et al., 2016). The decline in cognitive functioning in the cold have been explained by the discomfort caused by the cold exposure, resulting in difficulties in concentration. This “distraction theory” have been proposed by Muller et al. (2012), and later supported by studies of Taylor et al. (2015). Other studies have suggested that acute cold exposure may reduce levels of catecholamines or thyroid hormones, which in turn is associated with worse cognitive functioning (Shurtleff et al., 1994; Leppäluoto et al., 2005). Moderate cold exposure affects cognition function negatively through the mechanisms of distraction and both positively and negatively through the mechanism of arousal (increased vigilance in the cold) (Mäkinen, 2007). The study by Mäkinen (2007) proposes that especially simple cognitive tasks are adversely affected by cold, while more complex tasks may even improve in mild or moderate cold.

Occupations that require outdoor work in cold environments (Naesgaard et al., 2017) in combination with night- work face a combination of several interacting occupational health risk factors. Both night- and shift work are known to affect cognitive functioning and sleep negatively (Friborg et al., 2014). Shift workers commonly report poor sleep and decreased sleep quality (Harris, 2011). This increases the risk of human error (Akerstedt et al., 2007; Akerstedt, 2007) and can be a concurrent factor in explaining why many critical accidents are more prevalent at night (Mitler et al., 1988). The natural circadian rhythm in core body temperature results from the heat gain and heat loss mechanisms caused by the suprachiasmatic nuclei (SCN) in the hypothalamus, and regulates the natural lowering of core temperature at night (Panda, 2016). Working at night, when the body naturally regulates metabolism and temperature to a lower level, may be a contributing factor to the decline in cognitive functioning and especially vigilance at night.

To our knowledge no studies have previously investigated the combination of work at low ambient temperatures during night and the possible negative impact this may have on cognitive performance.

The objective of this study was to investigate night work at low ambient temperatures, and to what extent this will affect cognitive performance (short-term memory and reaction time), skin- and core temperature, thermal comfort, sleepiness, and cortisol. We hypothesized that cognitive performance is reduced at night compared with daytime and worsened when exposed to low ambient temperatures at night.

## Methods

### Test Subjects

Eleven male volunteer subjects were recruited into the study (age, 23 ± 2 years; mass, 81.4 ± 5.5 kg; height, 1.85 ± 0.04 cm; body mass index, 23.5 ± 2.1 kg ⋅ m^–2^; and body fat, 13.7 ± 3.2%). The subjects were non-smokers, had no sleep disturbances and had not flown between time zones in the week that preceded the trial. The subjects had a normal night’s sleep before the test and did not have any coffee, tea, cola soft drink or chocolate in the 2 h that preceded the test, or alcohol or tobacco 24 h before the test. All participants were informed about the aim of the study, the test protocol, and their rights to terminate their participation at any time in accordance with the 2013 Declaration of Helsinki before they provided written consent. All subjects underwent a doctor’s medical check-up in advance of the study. The exclusion criteria were previous cold-related injuries or Raynaud’s syndrome. The study was approved by the Committee for Medical and Health Research Ethics, Central Norway.

### Experimental Protocol

To familiarize themselves with the cognitive test battery and prevent a learning effect, the participants performed five pre-tests before participating in the tests. The participants then performed three main tests in a climatic chamber in the Work Physiology Laboratory at the Department of Health Research at SINTEF, Trondheim, Norway, in January and February 2017. Each test had a duration of 6 h and were performed during the night at –2 and 23^°^C ambient temperature and a control series at 23°C at daytime. The three series was fully randomized, and each participant accomplished the different tests and was their own control.

Upon arrival at the laboratory, the subjects were equipped with thermistors, a rectal probe and a heart-rate recorder. They were dressed in underwear and rested in a room with temperature of 22–23°C outside the climatic chamber. Two test subjects participated in each test. The subjects were allowed to drink water (2 cups *ad libitum*) and eat a banana or apple at 03:00 am (night test) or 11:00 am (day test). They were dressed in woolen underwear and protective outdoor work clothing and moved into the climatic chamber, where they performed identical repeated activities as follows; Time at day/night trials; 08:00/00:00 and every second hour; cognitive test, 08:00/00:00 and every hour; subjective score, 08:30/00:30 and every hour; treadmill (6 min, 4 km/h); 12:00/04:00 cortisol test.

### Measurements

Upon arrival at the laboratory, the height and weight (IDI; Mettler-Toledo, Albstadt, Germany) of the participants were recorded and the fat percentage was measured using a skinfold pinch (Harpenden Skinfold Caliper; Baty, United Kingdom). The amount of subcutaneous fat was measured on m. Biceps brachii, m. Triceps brachii, m. Subscapularis and the supra-iliac skinfold and calculated based on the formula given by Durnin and Womersley (1974).

To measure the body’s heat content and detect changes in body heat, continuous measurements of core and skin temperatures were performed using a rectal probe and thermistors (YSI 400; Yellow Springs Instruments, Yellow Springs, OH, United States), respectively, with an accuracy of ±0.15^°^C. The rectal probe was inserted to a depth of 10 cm from the spinal cord. Average skin temperature was calculated from the formula given by Ramanathan (1964), based on measurements from the chest, upper arm, thigh and leg.

The subjects were asked to evaluate their own thermal comfort and temperature sensation during the experiment. Thermal sensation was evaluated on a scale from –5 to 5 (extremely cold to extremely hot) and thermal comfort was evaluated on a scale of 1–4 (comfortable to very uncomfortable) (Nielsen et al., 1989).

Sleepiness was evaluated using the 9-step Karolinska Sleepiness Scale (KSS), which is based on a scoring from 1 = very awake to 9 = sleepy (fighting against sleep) (Akerstedt and Gillberg, 1990).

To test cognitive function (reaction time and short-term memory), the iPad-based system CANTAB Connect from Cambridge Cognition (Cambridge, United Kingdom) was used. The paired associates learning (PAL) and reaction time (RT) cognitive tests were performed. In the PAL test, boxes appear on the screen and are “opened” in a random order. One or more boxes contain a pattern. The patterns are then displayed at the center of the screen one at a time and the participant must select the box in which the pattern was originally placed. If the participant makes a mistake, the boxes are reordered, to remind the participant of the placement of the patterns. In the RT test, the subject holds a response button at the bottom of the screen. A series of five circular figures is presented above, and a yellow dot appears in one of the circles. The respondent must respond as quickly as possible and release the response button at the bottom of the screen and select the circle in which the dot appears. The test measures the average time required (in ms) for a subject to release the response button and select the yellow stimulus presented on the screen.

The cortisol test was administered at 4:00 am and at 12:00 pm on the respective night and day trials. All saliva analyses required at least 1 ml of saliva, which was collected in a test tube. Saliva production was not stimulated; rather, it formed naturally. No food was consumed during the 30 min that preceded sampling. Samples were stored immediately at –20^°^C. The number and time of samplings of the test subjects were recorded. The samples were analyzed by the Department of Medical Biochemistry, Laboratory Centre at St. Olav’s Hospital, Trondheim, Norway.

During the experiments, the following clothing was worn. Low ambient temperature (–2^°^C): woolen sweater, long underpants, wool jacket and pants, parka and trousers, headgear, balaclava and hood, winter shoes, gloves and wool socks. High ambient temperature (23^°^C): underwear, woolen sweater, long underpants, and wool socks.

### Statistical Analysis

Statistical analyses were performed using SPSS for Windows (v. 18.0; SPSS Inc., Chicago, IL, United States) and Microsoft Excel 2010 (Microsoft Corp., Redmond, WA, United States). Data were checked for normality using Kolmogorov–Smirnoff tests. The difference in cognitive performance response under the different ambient conditions was assessed by two-way analyses of variance (ANOVA) for repeated measures as previously done by Faerevik and Reinertsen (2003) A within group study design was used and all subjects were their own control. All cognitive performance data were tested for effects of time, ambient condition, and the interaction between these two. Time-dependent changes in rectal temperature and mean skin temperatures were also evaluated by two-way analyses of variance (ANOVA) for repeated measures. When ANOVA revealed a significant main effect, a contrast test was used as a *post hoc* test to locate the significant differences between the temperatures. Temperature data were analyzed every 10 min and cognitive measurements had four data points in the analysis. Subjective measurements were analyzed using Friedman’s non-parametric test for repeated measurements. Subjective measurements had seven data points in the analysis. Data are presented as the average and standard deviation. Significance was set at *P* < 0.05.

## Results

**FIGURE 1:**
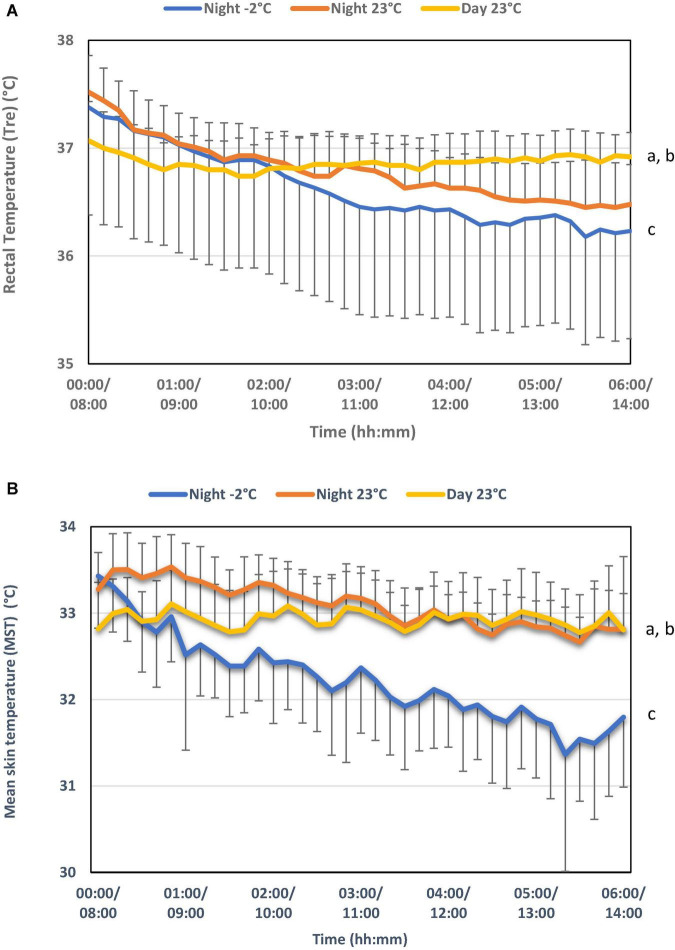
**(A)** Rectal temperature (T_re_) and **(B)** mean skin temperature (MST) for all exposures. a. A significant difference in the time dependent development of temperature between Night –2°C and Day 23°C for T_re_ (*p* = 0.001) and MST (*p* = 0.001). b. A significant difference in the time dependent development of temperature between Day 23°C and Night 23°C for T_re_ (*p* = 0.001) and MST (*p* = 0.001). c. A significant difference in the time dependent development of temperature between Night –2°C and Night 23°C for T_re_ (*p* = 0.026) and MST (*p* = 0.001). Data are presented as means ± SD (*n* = 11 for MST, *n* = 10 for T_re_, missing data for one person).

There was a significantly greater reduction in core temperature in the night trial performed at –2^°^C (from 37.4 to 36.2^°^C) compared with that performed at 23^°^C at night (from 37.5 to 36.5^°^C) (Anova GLM analysis within subjects’ effects: Temp × tid (*p* = 0.026, *df* = 6,224, *F* = 2,585) (Figure 1). In the day trial in the warm condition (23^°^C), a stable core temperature was maintained throughout the test, with a change from 37.0^°^C at the start to 36.9^°^C toward the end of the test. Both night trials (–2 and 23°C) had a significant greater reduction in core temperature compared to the day trial in 23°C [Anova GLM analysis within subjects effects: Night –2°C vs. Day 23°C: Temp × time (*p* = 0.001, *df* = 4,705, *F* = 29,728), Night23°C vs. Day 23°C: Temp × time (*p* = 0.001, *df* = 4,631, *F* = 20,389)].

**FIGURE 2:**
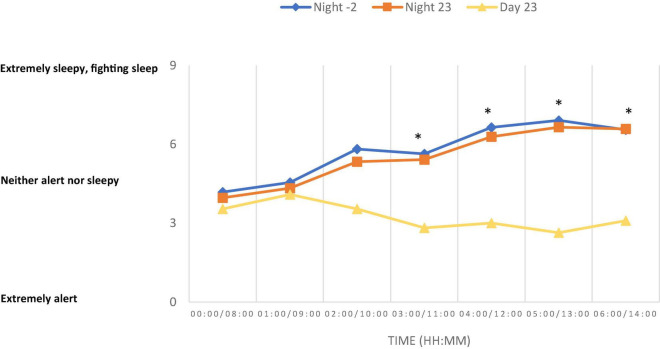
Sleepiness (Karolinska Sleepiness Scale). *Significant increase in sleepiness at night compared to and day at both temperatures (Night –2°C and Night 23°C). Data are presented as means (*n* = 11).

**FIGURE 3:**
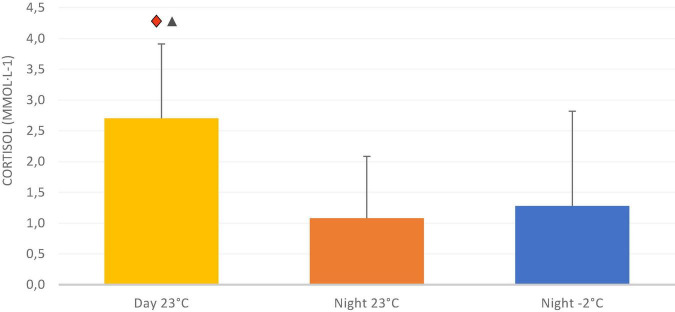
Cortisol in saliva at 04:00 am and at 12:00 pm under all exposures. (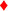) A significant difference between Day 23°C and Night 23°C and (▲) significant difference between Day 23°C and Night –2°C. Data are presented as means ± SD (*n* = 11).

The average skin temperature was significantly lower after exposure to –2^°^C compared with 23^°^C during both the night and day tests (Figure 1). The skin temperature was 33.4^°^C at the start of the night test and 32.5^°^C at the start of the day test, regardless of ambient temperature. The skin temperature was relatively stable throughout both tests at 23^°^C, but fell to 31.8^°^C in the night test at –2^°^C. During all trials, small variations (less than 0.5^°^C) in the average skin temperature were observed which is due to the variation between rest and easy work. The repeated work periods included in the test did not significantly affect core temperature. Thermal sensation of the body, feet, hands, and head showed that the subjects were significantly colder and more uncomfortable when exposed to –2^°^C compared with 23^°^C, regardless of whether it was night or day. There were no significant differences in thermal sensation or comfort during the night or day exposure to 23^°^C. There were no significant effects of either ambient temperature or day/night on reaction time or short-term memory during the experiments (Table 1). Sleepiness increased significantly throughout the night at both –2 and 23^°^C. During the day trial, there was significant less decrease in sleepiness over time compared with night trials (Figure 2). Cortisol levels were significantly lower during both the night trials compared with the daytime trial; however, there was no effect of ambient temperature on cortisol level at night (Figure 3).

**TABLE 1:** Cognitive parameters.

	00:00/08:00	02:00/10:00	04:00/12:00	06:00/14:00
**(1a) Reaction time in milliseconds**				
Night 23°C	141 ± 27	140 ± 22	136 ± 30	139 ± 20
Night −2°C	143 ± 21	150 ± 29	142 ± 24	141 ± 19
Day 23°C	141 ± 21	138 ± 22	137 ± 22	142 ± 24
**(1b) Short term memory for every other hour for all exposures.**				
Night 23°C	3.6 ± 2.8	3.4 ± 2.1	2.3 ± 2,0	3.8 ± 3.8
Night −2°C	2.9 ± 4.2	3.9 ± 4.2	4.5 ± 4.0	4.1 ± 4.9
Day 23°C	3.9 ± 3.2	2.5 ± 2.3	4.1 ± 4.2	2.5 ± 2.6

*Data are presented as means ± SD (n = 11).*

## Discussion

The objective of this study was to investigate the effect of night work at low ambient temperatures, and to what extent this will affect cognitive performance (short-term memory and reaction time), skin- and core temperature, thermal comfort, sleepiness, and cortisol. A control series was performed at day at 23°C.

The results for this study demonstrates a natural lowering of core temperature at night, as reported by Kräuchi (2002). During both night studies performed at –2 and 23^°^C, the core temperature were higher in the afternoon when arriving to the laboratory and decreased gradually during night, which is a well-known physiological response (Wright et al., 2002). A slight increase in heat production was attributed to the preparation performed before the experiment, followed by a return of the core temperature to normal levels as reported earlier (Wiggen et al., 2011). The change in skin temperature was also dependent on whether it was night or day, which can be explained by the natural diurnal variation in core temperature. Sleep usually occurs in the descending part of the core temperature curve, when the heat loss from the body is maximal (Kräuchi et al., 2000). This indicates a close relationship between sleeping and heat loss from the body. However, in this study the participants were not allowed to sleep, and this may have influenced the normal regulation of core temperature during night. A lower core- and skin temperature was observed at –2^°^C at night compared to 23^°^C (both night and daytime) with corresponding lower thermal sensation and comfort of the body, especially the hands and feet in –2^°^C.

Several studies have demonstrated impairment in cognitive performance (e.g., reaction time and short-term memory) upon exposure to low ambient temperatures (Stang and Wiener, 1970; Coleshaw et al., 1983; Palinkas, 2001). Physiological parameters, such as skin- and core temperature, are often used as indicators of reduction in cognitive performance. Reaction time is also among the parameters that are most affected during a simulated heat stress in pilots (Faerevik and Reinertsen, 2003). Thermal comfort can have a greater impact on cognitive performance compared to physiological parameters (Pilcher et al., 2002) since the discomfort by freezing will be able to distract the person and in this way reduce the reaction time and precision of the given the task (distraction theory). The results of our study suggest that neither low ambient temperatures nor night work influences short-term memory or reaction time. This is in contrasts with other studies in cold environments and must be considered in relation to the degree of thermal stress and that the subjects were protected by the clothing worn in the test. Muller et al. (2012) showed that several cognitive functions were reduced even at an ambient temperature of 10^°^C and that the reduced functions remained impaired over a heating period. The major difference between that study and our work was the clothing; Muller et al. (2012) dressed their subjects in shorts only before exposure to an ambient temperature of 10^°^C. This led to a significantly greater reduction in average skin temperature and thermal comfort compared with our study. Muller et al. (2012) reported an average skin temperature of about 22–23^°^C and a thermal sensation of cold. They emphasized that the exact physiological mechanisms that explain the reduction in cognitive performance observed during acute cold exposure is unclear but suggested that acute adaptations in the blood vessels in the brain caused by cold exposure could lead to cognitive dysfunction. Vasoconstriction has been detected in several parts of the circulatory system during cold exposure, but the specific relationship between skin cooling and the effect on the brain remains unclear (Muller et al., 2012). Adequate clothing will limit the acute effect of exposure to low ambient temperature, which minimizes the risk of decreased cognitive performance during work. Optimized clothing is one important factor for maintaining cognitive performance in cold environments (Taylor et al., 2015). In our study the subjects were exposed to significant ambient cold stress at night; but they did not experience any critically low skin temperatures (below 10^°^C) and subjective thermal comfort was between neutral and slightly cool throughout the test. The lack of more severe cold stress is partly explained by the protective clothing worn.

Sleepiness is commonly affected by exposure to night shifts and gradually increases beyond the night. In our laboratory study the subjects experienced significant sleepiness at the night trials, but we did not find any effect of sleepiness on cognitive parameters. This is in contrast to field studies that have demonstrated reduced cognitive performance at the end of 12 h night and day shifts (Kazemi et al., 2016). This study also showed that cognitive performance was reduced to a greater extent at night compared with day, as assessed using reaction time as a measurement parameter. Our findings did not support this study, one reason may be that our study only had a duration of 6 h. The subjects of Kazemi et al.’s (2016) study were all familiar with night work and reported being less sleepy (score of 5 on the KSS) than the participants in our study (score of 7 on the KSS), who were not used to night work. Our study simulated the first night of a nightshift, with an absence of adaptation to night work. In real-world working conditions, the daily rhythm will gradually adapt to the new work situation. A study from 2010 documented this adaptation over 7 days of 12 h shifts (Hansen et al., 2010). That study was performed on supply vessels in the North Sea, where vessel movements, noise and vibration also play a role and probably reduce the ability to adapt. In studies performed on oil platforms in the North Sea (Gibbs et al., 2002, 2007) similar adjustments were observed for 12 h shift work.

The level of cortisol measured in the saliva was significantly higher during daytime (at 12:00 pm) compared with night (at 4:00 am), but no effect of ambient temperature on cortisol levels was found. Day-to-day cortisol variations, as shown in this study, are well-documented in the literature, showing that the level of cortisol in the blood (and in the urine and saliva) usually rises and decreases in line with the daily rhythm. The cortisol level is highest at rising time (~8:00 am). This value then decreases somewhat and remains relatively stable throughout the day. In the evening, the values drop and reach a minimum around midnight (Turek and Zee, 1999). However, this pattern may change if a person is working irregularly (e.g., night shift) and sleeps at different times of the day. The participants in our study were recruited from a normal population and were informed before the study to sleep as normal as possible. Therefore, we consider that the cortisol level of the participants followed a normal daily variation.

Cortisol is secreted in response to various stressful situations and studies of the effects of short-term cold exposure have yielded conflicting results. In our study, no effect of ambient temperature on cortisol was found. This agrees with the conclusion of a study reported by Granberg (1995), which showed that cold stress does not increase cortisol secretion. However, other studies have found increased cortisol secretion after exposure to low ambient temperatures. A 2 h exposure to low temperatures (5–15^°^C) increased cortisol levels (Wagner et al., 1987), and the addition of physical stress and cold showers increased these levels further (Tikuisis et al., 1999). Some studies have also reported reduced (Wittert et al., 1992) or unchanged (Gerra et al., 1992) cortisol levels in response to cold exposure. As discussed earlier, the participants were dressed in warm winter clothes throughout the experiment and reported being comfortable or a little cool throughout the test period in the low ambient temperature at night. Probably, the exposure to low ambient temperatures was insufficient to increase cortisol secretion due to the protection provided by the clothing worn. Several other factors may affect the secretion of cortisol. It has been shown that both psychological and physical stresses under arctic conditions can be expected to activate the body’s response to stress, which may result in increased cortisol secretion. An increased level of cortisol was detected during an irregular trip with dog sledding in the Arctic, probably because of increased mental strain (Steine et al., 2003). Other expedition studies, however, found no increased cortisol values (Bishop et al., 2001). In our study, the only physical effort was easy walking on a treadmill six times for 6 min during the trial. Neither the cognitive tests nor physical work were major stressors, and they were performed in an identical manner during all trials.

### Study Limitations

CANTAB Connect is a well-documented and reliable test method; but we did only include two cognitive parameters in our test battery for practical reasons. Short-term memory and reaction time might not be the most sensitive cognitive parameter to temperature cold stress as there might be an arousal effect of the low temperatures. Therefore, we cannot exclude the possibility that the inclusion of other cognitive parameters would have uncovered stronger negative effects in this study. Even though a practical application of our findings is that adequate clothing is of importance to protect against cooling and hence maybe protected from declines in cognitive performance, it is also a study limitation. We could have designed the protocol more like Muller et al. (2012) to provoke a more severe impact of the low ambient temperatures on skin and core temperature. The reason we selected to use protective clothing in –2^°^C was to simulate a realistic scenario for outdoor workers in the cold by using one of the best clothing available for the workers.

## Conclusion

This study investigated how low ambient temperatures and night work affects some cognitive performance parameters. Lower skin- and core temperature was observed at night when exposed to low ambient temperature (–2°C), but there was no effect on short-term memory or reaction time. Increased sleepiness and lower cortisol levels were observed at night compared to daytime and was not influenced by low ambient temperature at night. The result from this study suggests that cognitive performance (short-term memory and reaction time) is not adversely affected by night work when exposed to low ambient temperatures if adequate protective clothing is worn.

## Data Availability Statement

The original contributions presented in the study are included in the article/supplementary material, further inquiries can be directed to the corresponding author.

## Ethics Statement

The studies involving human participants were reviewed and approved by the Committee for Medical and Health Research Ethics, Central Norway. The patients/participants provided their written informed consent to participate in this study.

## Author Contributions

HF, MS, and ØW: study conception and design. ØW and JH: sample and data acquisition. HF, MS, ØW, and JH: data analysis and drafting of the manuscript. HF and MS: project supervision. All authors contributed to the article and approved the submitted version.

## Conflict of Interest

The authors declare that the research was conducted in the absence of any commercial or financial relationships that could be construed as a potential conflict of interest.

## Publisher’s Note

All claims expressed in this article are solely those of the authors and do not necessarily represent those of their affiliated organizations, or those of the publisher, the editors and the reviewers. Any product that may be evaluated in this article, or claim that may be made by its manufacturer, is not guaranteed or endorsed by the publisher.

## References

[B1] Akerstedt T. (2007). Altered sleep/wake patterns and mental performance. *Physiol. Behav.* 90 209–218. 10.1016/j.physbeh.2006.09.007 17049569

[B2] Akerstedt T. Gillberg M. (1990). Subjective and objective sleepiness in the active individual. *Int. J. Neurosci.* 52 29–37. 10.3109/00207459008994241 2265922

[B3] Akerstedt T. Kecklund G. Gillberg M. (2007). Sleep and sleepiness in relation to stress and displaced work hours. *Physiol. Behav.* 92 250–255. 10.1016/j.physbeh.2007.05.044 17585960

[B4] Bishop S. L. Grobler L. C. Schjoll O. (2001). Relationship of psychological and physiological parameters during an Arctic ski expedition. *Acta Astronaut.* 49 261–270. 10.1016/s0094-5765(01)00104-7 11669115

[B5] Castellani J. W. Young A. J. (2016). Human physiological responses to cold exposure: acute responses and acclimatization to prolonged exposure. *Auton. Neurosci.* 196 63–74. 10.1016/j.autneu.2016.02.009 26924539

[B6] Coleshaw S. R. Van Someren R. N. Wolff A. H. Davis H. M. Keatinge W. R. (1983). Impaired memory registration and speed of reasoning caused by low body temperature. *J. Appl. Physiol.* 55 27–31. 10.1152/jappl.1983.55.1.27 6885583

[B7] Dai L. Kloog I. Coull B. A. Sparrow D. Spiro A. III Vokonas P. S. (2016). Cognitive function and short-term exposure to residential air temperature: a repeated measures study based on spatiotemporal estimates of temperature. *Environ. Res.* 150 446–451. 10.1016/j.envres.2016.06.036 27391696 PMC5003630

[B8] Durnin J. V. G. A. Womersley J. (1974). Body fat assessed from total body density and its estimation from skinfold thickness: measurements on 481 men and women aged from 16 to 72 Years Body fat assessed from total body density and its estimation from skinfold thickness : measurements on 481 men and women aged from 16 to 72 years. *Br. J. Nutr.* 32 77–97. 10.1079/BJN19740060 4843734

[B9] Faerevik H. Reinertsen R. E. (2003). Effects of wearing aircrew protective clothing on physiological and cognitive responses under various ambient conditions. *Ergonomics* 46 780–799. 10.1080/0014013031000085644 12745979

[B10] Friborg O. Rosenvinge J. H. Wynn R. Gradisar M. (2014). Sleep timing, chronotype, mood, and behavior at an Arctic latitude (69^°^N). *Sleep Med.* 15 798–807. 10.1016/j.sleep.2014.03.014 24933084

[B11] Gerra G. Volpi R. Delsignore R. Maninetti L. Caccavari R. Vourna S. (1992). Sex-related responses of beta-endorphin, ACTH, GH and PRL to cold exposure in humans. *Acta Endocrinol.* 126 24–28. 10.1530/acta.0.1260024 1310561

[B12] Gibbs M. Hampton S. Morgan L. Arendt J. (2002). Adaptation of the circadian rhythm of 6-sulphatoxymelatonin to a shift schedule of seven nights followed by seven days in offshore oil installation workers. *Neurosci. Lett.* 325 91–94. 10.1016/s0304-3940(02)00247-1 12044629

[B13] Gibbs M. Hampton S. Morgan L. Arendt J. (2007). Predicting circadian response to abrupt phase shift: 6-sulphatoxymelatonin rhythms in rotating shift workers offshore. *J. Biol. Rhythms* 22 368–370. 10.1177/0748730407302843 17660453

[B14] Granberg P. O. (1995). Human endocrine responses to the cold. *Arctic Med. Res.* 54 91–103. 7639891

[B15] Hancock P. A. Ross J. M. Szalma J. L. (2007). A meta-analysis of performance response under thermal stressors. *Hum. Factors* 49 851–877. 10.1518/001872007x230226 17915603

[B16] Hansen J. H. Geving I. H. Reinertsen R. E. (2010). Adaptation rate of 6-sulfatoxymelatonin and cognitive performance in offshore fleet shift workers: a field study. *Int. Arch. Occup. Environ. Health* 83 607–615. 10.1007/s00420-010-0547-x 20499083

[B17] Harris A. (2011). *Adaptation and health in extreme and isolated environments: from 78°N to 75°S.* Ph.D. thesis. Norway: University of Bergen.

[B18] Kazemi R. Haidarimoghadam R. Motamedzadeh M. Golmohamadi R. Soltanian A. Zoghipaydar M. R. (2016). Effects of Shift Work on Cognitive Performance, Sleep Quality, and Sleepiness among Petrochemical Control Room Operators. *J. Circadian Rhythms* 14:1. 10.5334/jcr.134 27103934 PMC4834749

[B19] Kräuchi K. (2002). How is the circadian rhythm of core body temperature regulated? *Clin. Auton. Res.* 12 147–149. 10.1007/s10286-002-0043-9 12269545

[B20] Kräuchi K. Cajochen C. Werth E. Wirz-Justice A. (2000). Functional link between distal vasodilation and sleep-onset latency? *Am. J. Physiol. Regul. Integr. Comp. Physiol.* 278 R741–R748. 10.1152/ajpregu.2000.278.3.R741 10712296

[B21] Leppäluoto J. Pääkkönen T. Korhonen I. Hassi J. (2005). Pituitary and autonomic responses to cold exposures in man. *Acta Physiol. Scand.* 184 255–264. 10.1111/j.1365-201X.2005.01464.x 16026418

[B22] Mäkinen T. M. (2007). Human cold exposure, adaptation, and performance in high latitude environments. *Am. J. Hum. Biol.* 19 155–164. 10.1002/ajhb.20627 17286263

[B23] Mitler M. M. Carskadon M. A. Czeisler C. A. Dement W. C. Dinges D. F. Graeber R. C. (1988). Catastrophes, sleep, and public policy: consensus report. *Sleep* 11 100–109. 10.1093/sleep/11.1.100 3283909 PMC2517096

[B24] Muller M. D. Gunstad J. Alosco M. L. Miller L. A. Updegraff J. Spitznagel M. B. (2012). Acute cold exposure and cognitive function: evidence for sustained impairment. *Ergonomics* 55 792–798. 10.1080/00140139.2012.665497 22506538 PMC3375336

[B25] Naesgaard O. P. Storholmen T. C. B. Wiggen ØN. Reitan J. (2017). A user-centred design process of new cold-protective clothing for offshore petroleum workers operating in the Barents Sea. *Ind. Health* 55 564–574. 10.2486/indhealth.2017-0127 29046494 PMC5718777

[B26] Nielsen R. Gavhed D. C. E. Nilsson H. (1989). Thermal function of a clothing ensemble during work: dependency on inner clothing layer fit. *Ergonomics* 32 1581–1594. 10.1080/00140138908966927 2634560

[B27] Palinkas L. A. (2001). Mental and cognitive performance in the cold. *Int. J. Circumpolar Health* 60 430–439. 11590885

[B28] Panda S. (2016). Circadian physiology of metabolism. *Science* 354 1008–1015. 10.1126/science.aah4967 27885007 PMC7261592

[B29] Pilcher J. J. Nadler E. Busch C. (2002). Effects of hot and cold temperature exposure on performance: a meta-analytic review. *Ergonomics* 45 682–698. 10.1080/00140130210158419 12437852

[B30] Ramanathan N. L. (1964). A new weighting system for mean surface temperature of the human body. *J. Appl. Physiol.* 19 531–533. 10.1152/jappl.1964.19.3.531 14173555

[B31] Ramsey J. D. (1995). Task performance in heat: a review. *Ergonomics* 38 154–165. 10.1080/00140139508925092 7875117

[B32] Shurtleff D. Thomas J. R. Schrot J. Kowalski K. Harford R. (1994). Tyrosine reverses a cold-induced working memory deficit in humans. *Pharmacol. Biochem. Behav.* 47 935–941. 10.1016/0091-3057(94)90299-2 8029265

[B33] Stang P. R. Wiener E. L. (1970). Diver performance in cold water. *Hum. Factors* 12 391–399. 5460268 10.1177/001872087001200405

[B34] Steine K. Røseth A. G. Sandbaek G. Murison R. Slagsvold C. E. Keller A. (2003). [Increased cortisol levels, frostbite and effects on the muscles and skeleton during extreme polar conditions]. *Tidsskr. Nor. Laegeforen.* 123 3529–3532. 14691491

[B35] Taylor L. Watkins S. L. Marshall H. Dascombe B. J. Foster J. (2015). The Impact of Different Environmental Conditions on Cognitive Function: a Focused Review. *Front. Physiol.* 6:372. 10.3389/fphys.2015.00372 26779029 PMC4701920

[B36] Tikuisis P. Ducharme M. B. Moroz D. Jacobs I. (1999). Physiological responses of exercised-fatigued individuals exposed to wet-cold conditions. *J. Appl. Physiol.* 86 1319–1328. 10.1152/jappl.1999.86.4.1319 10194218

[B37] Turek F. W. Zee P. C. (1999). “Lung biology in health and disease,” in *Regulation of sleep and circadian rhythms*, ed (New York: Marcel Dekker), 133

[B38] Wagner J. A. Horvath S. M. Kitagawa K. Bolduan N. W. (1987). Comparisons of blood and urinary responses to cold exposures in young and older men and women. *J. Gerontol.* 42 173–179. 10.1093/geronj/42.2.173 3819343

[B39] Wiggen ØN. Heen S. Færevik H. Reinertsen R. E. (2011). Effect of cold conditions on manual performance while wearing petroleum industry protective clothing. *Ind. Health* 49 443–451. 10.2486/indhealth.ms1236 21697624

[B40] Wittert G. A. Or H. K. Livesey J. H. Richards A. M. Donald R. A. Espiner E. A. (1992). Vasopressin, corticotrophin-releasing factor, and pituitary adrenal responses to acute cold stress in normal humans. *J. Clin. Endocrinol. Metab.* 75 750–755. 10.1210/jcem.75.3.1517364 1517364

[B41] Wright K. P. Hull J. T. Czeisler C. A. (2002). Relationship between alertness, performance, and body temperature in humans. *Am. J. Physiol. Regul. Integr. Comp. Physiol.* 283 R1370–R1377. 10.1152/ajpregu.00205.2002 12388468

